# Inconclusive evidence that breathing shapes pupil dynamics in humans: a systematic review

**DOI:** 10.1007/s00424-022-02729-0

**Published:** 2022-07-25

**Authors:** Martin Schaefer, Sylvia Edwards, Frans Nordén, Johan N. Lundström, Artin Arshamian

**Affiliations:** 1 Department of Clinical Neuroscience, Karolinska Institutet, 17177 Stockholm, Sweden; 2 Monell Chemical Senses Center, Philadelphia, PA 19104 USA; 3 Stockholm University Brain Imaging Centre, Stockholm University, 11415 Stockholm, Sweden

**Keywords:** Breathing, Respiration, Phase, Pupil dynamics, Pupil size, Hippus

## Abstract

More than 50 years ago, it was proposed that breathing shapes pupil dynamics. This widespread idea is also the general understanding currently. However, there has been no attempt at synthesizing the progress on this topic since. We therefore conducted a systematic review of the literature on how breathing affects pupil dynamics in humans. We assessed the effect of breathing phase, depth, rate, and route (nose/mouth). We followed the Preferred Reporting Items for Systematic Reviews and Meta-Analyses (PRISMA) guidelines, and conducted a systematic search of the scientific literature databases MEDLINE, Web of Science, and PsycInfo in November 2021. Thirty-one studies were included in the final analyses, and their quality was assessed with QualSyst. The study findings were summarized in a descriptive manner, and the strength of the evidence for each parameter was estimated following the Grading of Recommendations Assessment, Development, and Evaluation (GRADE) approach. The effect of breathing phase on pupil dynamics was rated as “low” (6 studies). The effect of breathing depth and breathing rate (6 and 20 studies respectively) were rated as “very low”. Breathing route was not investigated by any of the included studies. Overall, we show that there is, at best, inconclusive evidence for an effect of breathing on pupil dynamics in humans. Finally, we suggest some possible confounders to be considered, and outstanding questions that need to be addressed, to answer this fundamental question. Trial registration: This systematic review has been registered in the international prospective register of systematic reviews (PROSPERO) under the registration number: CRD42022285044.

**Supplementary Information:**

The online version contains supplementary material available at 10.1007/s00424-022-02729-0.

## Introduction

The rhythmic activities of both breathing and pupil function can be observed with the naked eye. However, while the output of these rhythms is peripheral, both originate from the central nervous system [41, 48]. More than 50 years ago, it was suggested that breathing rhythm affects the pupil rhythm [8, 18]. Specifically, that inhalation increased pupil size by two independent pathways; one of central origin, and one emerging from changes in blood pressure via sinoaortic baroreceptors [8]. While this initial finding has been cited many times, to the best of our knowledge, no effort has been made into synthesizing the progress on this topic since. This is unfortunate, because both rhythms have—for different, yet complementary, reasons—been considered to be important factors that should be measured when studying brain and behavior [68, 69].

### Why study breathing and pupil function?

Breathing is one of the fundamental rhythms of life, but its effects stretch far beyond basic oxygenation [22, 31, 48, 69]. A series of recent neurophysiological studies with humans and non-human animals have demonstrated that breathing rhythmically activates core brain structures that control both perceptual and cognitive functions [7, 22, 27, 29–33, 37, 38, 45, 49, 54, 61, 76, 78]. Specifically, both the phase of the breathing cycle (i.e., if it is inhalation or exhalation), as well as the route of the inhalation (i.e., if it is through the nose or mouth) are important factors shaping brain activity and behavior [2, 22, 27, 54, 76]. For example, humans have a tendency to spontaneously inhale when they first engage in a cognitive task, an act that impacts brain activation and enhances performance compared to exhalation [54]. Similarly, the inhalation phase in rodents rhythmically regulates the whole brain [27, 69]. Beside the phase, the route of breathing is critical for these observations; breathing through the nose seems to rhythmically activate core brain structures fundamental for memory functions more directly than mouth breathing [49, 76]. This might be explained by the fact that mammalian olfactory sensory neurons detect mechanical pressure caused by airflow in the nostrils, even when there is no odor [19]. The airflow information reach the olfactory bulb [31] and generate oscillations transmitted to the piriform cortex, and from there propagate further downstream to the hippocampal formation—an area critical for memory function [7, 22, 29–31, 37, 38, 49, 61, 78]. Importantly, parallel to the olfactory bulb oscillator, there is also the main breathing oscillator in the brainstem—the pre-Bötzinger complex—which controls both nose and mouth breathing, and is therefore active during both [48]. Notably, during inhalation, the brainstem oscillator also impacts brain function and behavior, such as memory and arousal, independent of breathing route [48], but to a lesser extent than when it is combined with projections from the olfactory bulb [27]. Moreover, different patterns of breathing—for example deep diaphragmatic vs. more shallow breathing—differentially impact attention and stress parameters [40].

Whereas breathing shapes global brain activity, pupil dynamics reflect it [68]. While the field of cognitive neuroscience has largely ignored respiration as a useful measure [63] pupillometry has been extensively used [16]. Pupillometry has been used based on the knowledge that pupil dynamics are not only the result of the amount of light entering the eye, but that the pupils may change as a direct function of internal states [16]. In fact, changes in pupil diameter have been observed for most cognitive processes in humans (e.g., mental effort, attention, decision-making, and memory processes, to name some) [16].

Given these facts, it is critical to evaluate the evidence for any potential interaction between these two rhythms.

The aim of this review is to identify to what degree, and in which ways breathing affects pupil dynamics in humans. To limit the scope of the review, we restricted the systematic search to only include scientific literature on studies assessing effects in human participants. Additionally, we only included studies using healthy adults who were not taking any drugs. The latter, because we are interested in the normal functioning of breathing influences on pupil dynamics. However, we included studies where the interplay between breathing and pupil dynamics was not the main focus of the research question to gain a thorough understanding of the complete literature.

The breathing parameters we considered were breathing phase, breathing rate, breathing depth and breathing route. The pupil parameters we considered were, pupil diameter, fluctuations in pupil diameter, pupillary unrest (hippus), and pupillary light reflex dynamics.

## Methods

### Information sources and search strategy

This systematic review followed the Preferred Reporting Items for Systematic Reviews and Meta-Analyses (PRISMA) guidelines consisting of a 27-item checklist which is outlined in Supplementary Table 1 [53]. The preregistered protocol of this systematic review can be found in the PROSPERO database, an international prospective register for systematic reviews, with ID CRD42022285044 (https://www.crd.york.ac.uk/prospero/).

A systematic search of the scientific literature databases MEDLINE, Web of Science, and PsycInfo was performed in November 2021 by author MS with the help of university librarians. The complete search strategy for each database can be found in the Supplementary Tables 2, 3, and 4. The search strategy was validated by correctly including six articles that had been identified as relevant before initiating the systematic search [18, 43, 47, 50, 65, 75].

### Exclusion and inclusion of articles

Studies identified in the literature search were eligible for inclusion if they were conducted on healthy human subjects and contained simultaneous measures of both breathing and pupil size. Studies identified from the literature search were excluded if.

- The participants were younger than 18
- The participants were taking drugs/medication or had a chronic or acute medical condition (healthy control groups were, however, included)
- Breathing and pupil size were simultaneously measured, but not reported
- The article was in a language other than English, German, Dutch, or Swedish (languages the authors are native speakers of)
- The article did not present original data
- The article was not available in full text

These broad and thorough inclusion criteria allowed us to identify a wide range of studies going from “resting-state” to task-specific paradigms (e.g., in the form of emotional, perceptual, and cognitive experiments). This meant that we also could compare any potential effect of the breathing-pupil relationship as a function of the paradigm at hand.

The identified articles were independently checked by two pairs of authors, where each pair reviewed half of the articles. Titles and abstracts were screened and studies were removed for not meeting the inclusion criteria, or because they were duplicates that the automatic deduplication removal process had missed. If the two reviewers' initial and independent assessment differed on whether an article should be included or not, it was resolved by discussion to reach a consensus. The articles written in German were an exception, as only one author was fluent in German. However, relevant information was summarized in English and inclusion or exclusion of these articles was discussed with at least one other independent reviewer. To keep track of inclusion and exclusion of the articles, we used the web-app Rayyan [52]. Articles remaining after the initial screening were assessed in detail in the same manner, where the same reviewers read the articles they had initially screened. If consensus on details could not be reached, the other reviewer pair was consulted to reach a final decision.

### Data extraction and management

We categorized the studies according to breathing parameters:

- Breathing phase
- Breathing depth
- Breathing rate
- Breathing route

Articles not containing any of the outcomes listed above are discussed separately. Data extraction was done independently by two research pairs per article and discrepancies were solved through discussion. Data extraction from German studies was done in the same way as the study assessment.

### Quality of evidence assessment

To assess the quality of the included studies, we used QualSyst, a tool developed for evaluating the internal validity of quantitative studies of various methodologies [18]. This tool includes 14 questions, answered with 0 (not at all), 1 (partially), 2 (yes), or NA (not applicable) (see Supplementary Table 5). The questions directly assessed potential bias such as sample size and appropriate design and analysis. It also evaluates factors relating to broader aspects of quality, such as how well the research question is described and whether the conclusions are supported by the results. A final overall score of quality (ranging from 0 to 1) is calculated by dividing the total score by the maximum possible score (i.e., excluding NAs).

Some of the questions in QualSyst assess appropriateness of study design and conclusions. The answer to these questions depends largely on whether the study set out to answer the question we were interested in. For this reason, in cases where assessing the relationship of breathing and the pupil was not the aim of the study we completed the quality assessment twice; once for the intended question of the paper, and once for our question. In the latter case, we left out questions 1 (question/objective sufficiently described?) in the QualSyst questionnaire and only included question 14 (conclusions supported by the results?) if relevant.

The quality assessment was carried out by two authors independently where the two pairs of authors assessed the same papers they had already assessed with regards to the inclusion criteria. Results for all individual questions of the independent quality assessments were discussed within each reviewer pair to reach an agreement on the score. Thus, the overall score was obtained through adjusting it based on discussion, rather than taking an average of the two results.

### Synthesis methods

To get an overview of the included studies, we categorized them based on three criteria. First, based on the breathing and pupil variables they measured. Second, on what kind of stimuli or task they used and, third, on whether an interaction between breathing and pupil measures (i.e., if breathing affected pupil dynamics) was a main study outcome (see Table 1).

**Table 1:** Study characteristics

Study	Participants	Breathing variable	Pupil variables	Stimuli/task	Breathing/pupil main study outcome
Al Abdi et al. 2018	58 (26 males, age 21.5, range 19–25)	Breathing rate	Pupil size	Math tasks with 3 levels of difficulty	No
Aue et al. 2016	45 (10 males, age 23.5 ± 4.1)	Breathing rate	Pupil size	Visual search task with three types of expectancy cues and birds and spiders as search targets	No
Babo-Rebelo et al. 2016	16 (8 males, age 24.1 ± 0.6)	Breathing rate and phase	Pupil size	Thought-sampling task	No
Bouma and Baghuis, 1971	‘some 20 subjects’	Breathing rate	Pupil size and hippus	Light stimuli, to either eye. Homogenous, patterns, different times, different distances, auditory stimuli, arithmetic tasks	Yes
Calcagnini et al. 2000	10 (age 24–28)	Breathing rate	Pupil size	Tone to cue breathing rate, head-up tilt 70°	Yes
D’Agostini et al. 2021	71 (16 males, age 23.3, range 18–35)	Breathing rate	Pupil size	Transcutaneous auricular vagus nerve stimulation	No
Daum and Fry, 1981	3 (age 22–24)	Breathing rate	Pupillary unrest and pupil size fluctuations	Continuous light stimulus	Yes
Debnath et al. 2021	21 (16 males, age 29.9 ± 6.5)	Breathing rate	Pupil size	Valsalva maneuvers, deep breathing	No
Gavriysky, 1991	19 (age 24.4)	Hyperventilation	Pupillary light reflex dynamics	Light stimulus and hyperventilation	Yes
Golenhofen and Petrányi, 1967	10 (6 males, age 20–34)	Breathing frequency and phase	Pupillary light reflex dynamics	Light flashes of 100 lx intensity and 10 ms duration	Yes
He et al. 2019	37 (18 males, age 26.4, range 20–35)	Breathing rate and depth	Pupil size	Driving simulation task, and n-back tasks	No
Jewell, 2017	354 (208 males)	Respiration line length	Pupil size	Relevant comparison test	No
Kaulen et al. 1979	17 (age 31, range 19–62)	Breathing phase	Pupillary light reflex dynamics	Light pulses of varying duration	Yes
Lindsley and Sassaman, 1938	1 (middle aged male)	Breathing rate and depth	Pupil size	Voluntary erection of body hair	No
Lyytinen, 1984	112 (all males, age 17–22)	Breathing cycle length and abnormal breathing cycles	Pupil size	Slides with patterns, a tone (of 70 dB intensity and 400 Hz frequency), a loud tone (of 87 dB and of 310 Hz frequency), an electric shock, arithmetic, memory and motor tasks	No
Mauri et al. 2011	30 (age 22 ± 2.1)	Breathing cycle length	Pupil size	Relaxation block of viewing panorama pictures, using Facebook block, stress block consisting of stroop task and arithmetical task	No
Melnychuk et al. 2018	14 (8 males, age 29 ± 7.7)	Breathing phase	Pupil size phase	Rest and visual oddball task	No
Melnychuk et al. 2021	13 (5 males, age 22–56)	Breathing phase	Pupil size	None	Yes
Murata and Iwase, 2000	10 (all males, age 20–23)	Breathing	Pupil size	Sternberg memory search task, and arithmetic task	No
Nakamura et al. 2019	14 (6 males, age 22.7 ± 0.8)	Breathing rate and phase	Pupil size	Figure memorization and recognition task	Yes
Ohtsuka et al. 1988	6 (age 23–40)	Breathing volume	Pupil size fluctuations	None	Yes
Onorati et al. 2013	13 (students)	Breathing rate	Pupil size	Recalling of happy, angry, and sad emotional events	No
Rebollo et al. 2021	29 (9 males, age 26.0 ± 4.9)	Breathing rate	Pupil size	210 pictures consisting of high-appeal food, low-appeal food, and non-food objects, extracted from the extended Salzburg food picture database	No
Rendon-Velez et al. 2016	54 (46 males age 28.5 ± 4.3, 8 females, age 27.0 ± 2.9)	Breathing rate and amplitude	Pupil size	A driving simulator task performed with and without time pressure	No
Robiner, 1990	40 (20 males, age 32.0 ± 5.1)	Breathing rate	Pupil size	Immersion in whirlpools and warm baths	No
Schumann et al. 2017	29 (8 males, age 36.9 ± 12.5, healthy controls)	Breathing rate	Pupil size and pupillary unrest index	None	No
Schumann et al. 2020	83 (24 males, age 23 ± 2) Deep breathing condition: 26 (15 males, age 37 ± 13)	Breathing rate, cycle length, volume	Pupil size and pupillary unrest index	An audio track of deep ventilatory noises	Yes
Schumann et al. 2015	29 (21 males, age 36 ± 13)	Breathing rate and cycle length	Pupil size and pupillary unrest index	None	Yes
Wöllner et al. 2018	46 (25 males, age 23.6 ± 4.3)	Breathing rate	Pupil size	Video excerpts of commercial films, dance, and sports footage. Videos were presented either in original slow motion or in adapted real-time motion. Videos were shown with and without music	No
Yoshida et al. 1994	4 (age 22–24)	Breathing depth and rate	Pupil size fluctuations	Fixation cross	Yes
Zénon, 2017	5 (4 males, age 23–38)	Breathing signal	Pupil size	None	No

Information about the included studies is summarized by listing participant age and gender, the reported breathing and pupil variables, the employed tasks and stimuli, and whether the main aim of the study was to investigate breathing and pupil dynamics

After identifying the main variables, commonalities between the different studies were visualized using an additional table showing the variables of interest present in each study (Table 2). Our predetermined main study outcomes for this review were the influence of breathing phase on pupil dynamics, with additional outcomes being changes in pupil dynamics depending on breathing depth, rate, and route. However, we did not find any studies that investigated the effects of breathing route on pupil dynamics and thus, this outcome measure could not be assessed. This left us with three outcome measures—*phase*, *depth*, and *rate*—that were then used to categorize the included articles. If studies included multiple measures, they were included in each of the listed parameters. The strength of evidence for each individual breathing parameter’s effect on pupil dynamics was quantified to provide an overview of the available literature. However, as specified in preregistration, a full statistical meta-analysis was beyond the scope of this systematic review. To this end, we plotted the studies measuring a certain breathing parameter based on their sample size, mean quality assessment score, whether their results were based on statistics, whether their main study outcome was breathing and pupil dynamics, and whether they found evidence that a breathing variable of interest affected pupil dynamics (Figs. 2, 3, 4, and 5). Figures 2, 3, 4, and 5 were created in R with the ggplot2 package and were aesthetically modified in Inkscape [23, 57, 73].

**Table 2:**
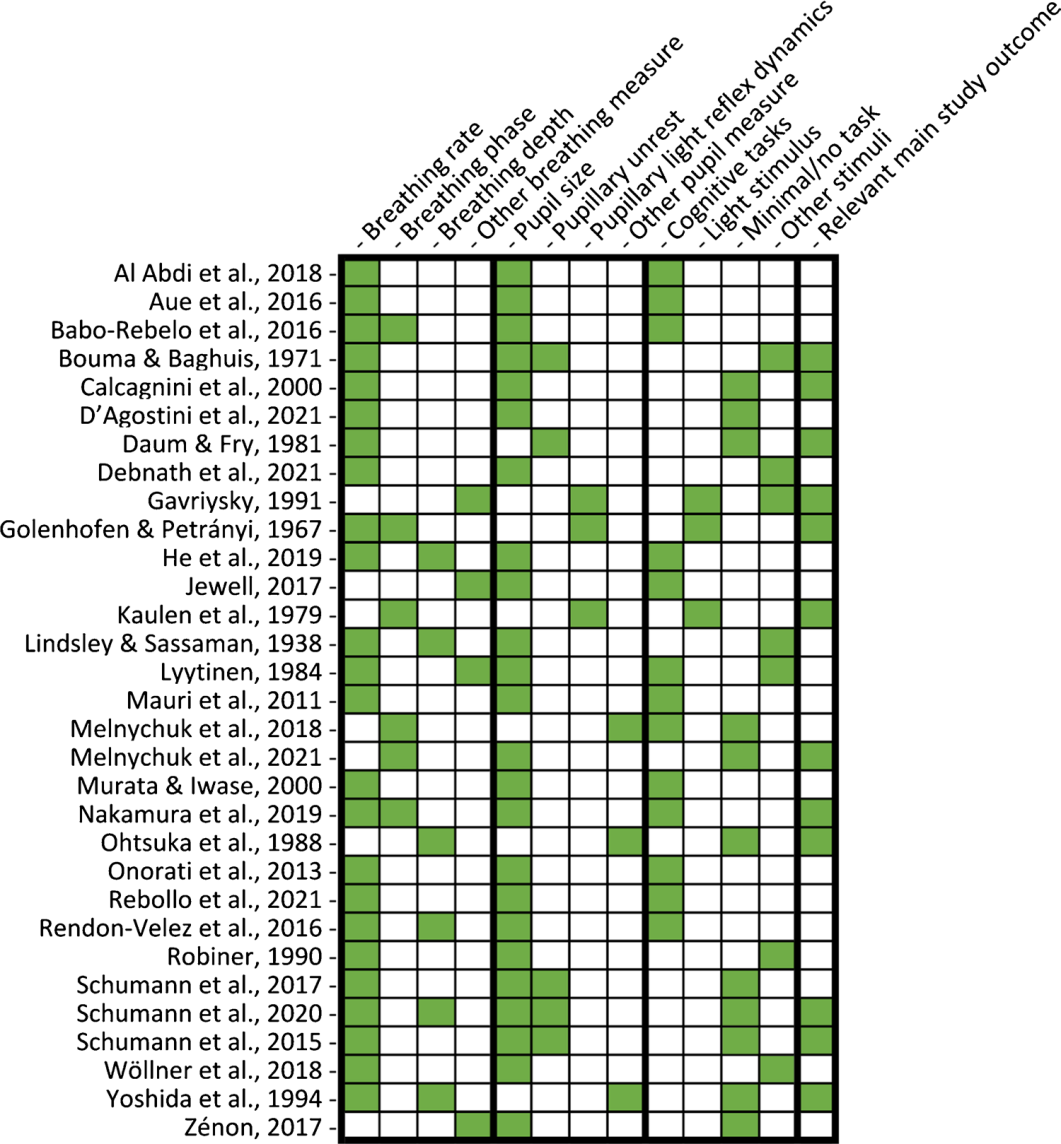
Study overview

The *x*-axis lists a variety of study characteristics, and the y-axis lists all studies included in this systematic review. A green bar marks the presence of a study characteristic in the study listed on the *y*-axis, whereas a white bar marks the absence of a study characteristic

### Certainty assessment

To assess the strength of the evidence for how the three main parameters of breathing (i.e., phase, depth, and rate) affected pupil dynamics, we followed the Grading of Recommendations Assessment, Development, and Evaluation (GRADE) approach [20].

The GRADE approach defines four levels of evidence, whilst recognizing that this discrete categorization is somewhat arbitrary:

- High—“We are very confident that the true effect lies close to that of the estimate of the effect.”
- Moderate— “We are moderately confident in the effect estimate: The true effect is likely to be close to the estimate of the effect, but there is a possibility that it is substantially different”
- Low—“Our confidence in the effect estimate is limited: The true effect may be substantially different from the estimate of the effect.”
- Very low—“We have very little confidence in the effect estimate: The true effect is likely to be substantially different from the estimate of effect”

The body of evidence was assessed for each outcome based on the design of the included studies and assigned one of the above categories. The GRADE approach then requires a systematic assessment of whether the evidence should be downgraded based on five criteria (study limitations/risk of bias, inconsistency of results, indirectness of evidence, imprecision, and publication bias), or upgraded based on three criteria (large magnitude of an effect, dose–response gradient, and effect of plausible residual confounding). However, because GRADE is a tool mainly developed for randomized control trials (RCT), we made minor adaptations to make the tool more applicable to the types of studies evaluated here.

Initial GRADE scoring of the study designs was based on judgement of the overall quality, which considered what degree of experimental control there was over the breathing variables, guided by results from the quality assessment. GRADE scoring then followed the original method, with some modification to the imprecision criterion, which normally would be based partially on confidence intervals. However, confidence intervals were often missing in the reported results for our studies. In this case, as well as when confidence intervals were present, we also considered stated *p* values, and whether they had used any statistical analyses (many studies did not), and or had a sufficient sample size. The final GRADE criterion, publication bias, was also left out due to the poor reporting of statistics in the articles, thereby making it difficult to estimate this risk. Whether or not to adjust the GRADE score in each instance was based on the reviewers’ evaluation of whether any of the studies met the given criteria, how many, and how influential these studies were on the outcome results. Because many of the relevant limitations had already been assessed with the quality assessment, we used the ratings of some of these questions to aid the GRADE scoring process. For example, ratings for sample size were considered under the imprecision criteria, and questions on measurement definition and control of confounding variables were considered under the risk of bias criterion. More details about the decision process can be found in the Supplementary materials (see "Detailed GRADE assessment”).

GRADE scoring for all the selected articles was done independently by three of the reviewers. Inconsistencies in the GRADE scoring and subsequent up/down grading was resolved in each instance through discussion among raters. The main result from the certainty assessment is presented in Table 3 and from the sub-categories in Figs. 2, 3, 4, and 5.

**Table 3:** Certainty of evidence as assessed by GRADE

Breathing parameter	Study design	Risk of bias	Inconsistency of results	Indirectness of evidence	Imprecision (uncertainty of results)	Total sample size across all studies	GRADE
Phase	Moderate	OK	OK	OK	↓	84 (6)	Low
Depth	Moderate	OK	↓	↓	↓	145 (6)	Very low
Rate direct	Moderate	OK	↓	OK	↓	188 (8)	Very low
Rate indirect	Moderate	OK	↓	↓	↓	368 (12)	Very low

The columns represent the categories given in the GRADE handbook and the arrows represent up- or downgrading [67]. Assessment of study design took account of the study’s quality assessment score. Risk of bias mainly evaluated confounding factors and how measurements were taken. The inconsistency category dealt with the incoherence in results between studies, and indirectness with whether pupil and breathing was the main study outcome. Imprecision dealt with sample size and application of statistical methods. Instead of assessing publication bias we show the total number of participants and number of studies for each study outcome in the seventh column. The GRADE column shows the final GRADE score for the evidence of each study outcome

## Results

### Study selection and characteristics

**Fig. 1:**
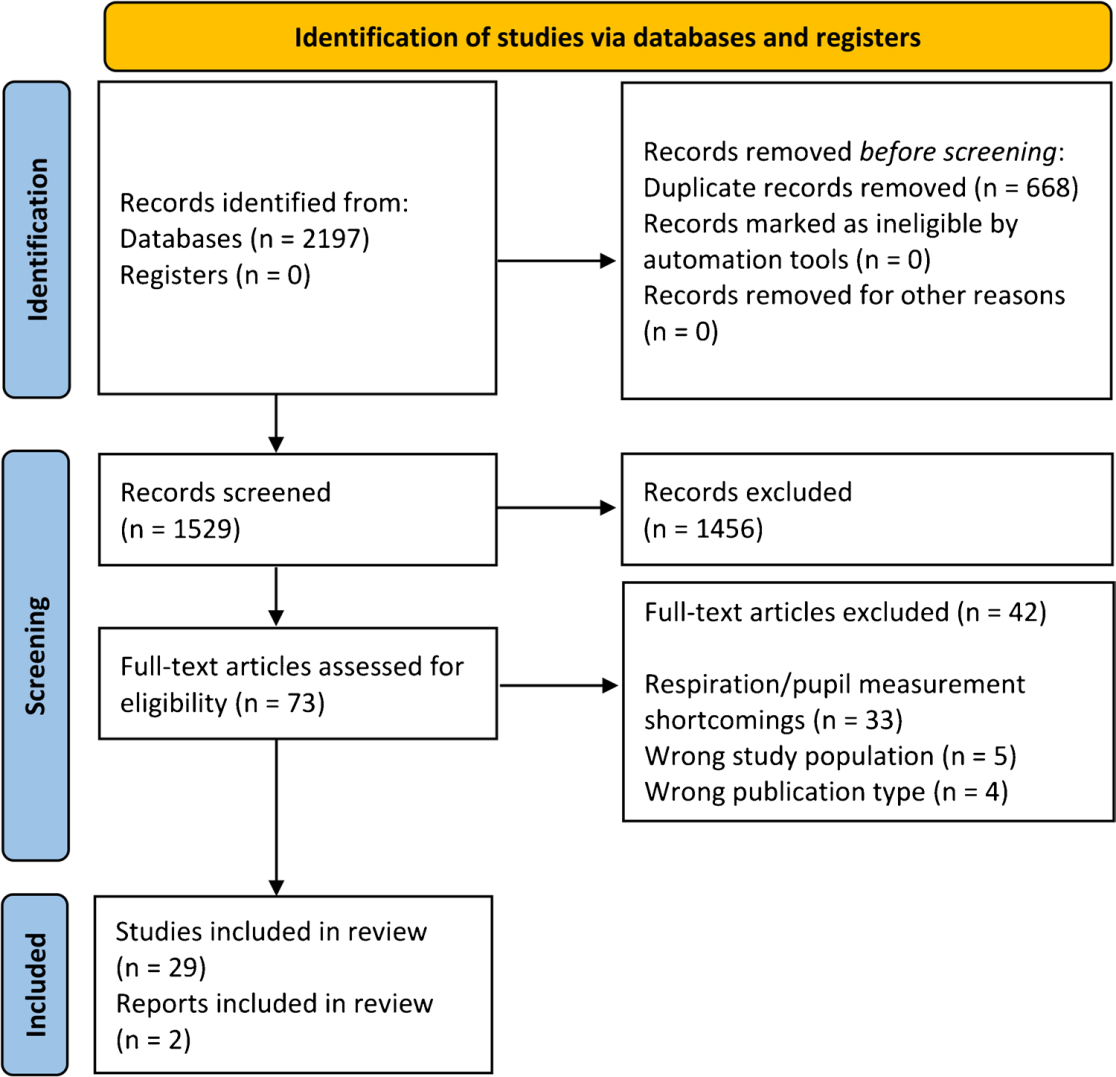
Flow diagram of study selection process. The included studies refer to journal articles, whereas the reports refer to academic theses

The literature search resulted in 2197 studies. After removal of 668 duplicates, we screened the remaining 1529 studies, selected 73 studies for full-text review, and excluded another 42 articles that were found to not meet the criteria. This resulted in 31 studies being included in the final review stage (see Fig. 1).

Three studies initially met our inclusion criteria but were later excluded [4, 21, 23]. These studies measured both breathing and pupil, however, the presentation of their data made them impossible to use in any meaningful way.

Table 1 presents a detailed overview of the study characteristics for the 31 studies (i.e., participants, breathing and pupil variables, stimuli, and whether the main study outcome was breathing and pupil dynamics). A concise overview is presented in Table 2.

### Study characteristics

#### Risk of bias in studies

The results of the risk of bias assessment of the individual studies are reported in Supplementary Tables 6 and 7.

### Results of synthesis

#### Breathing phase

The main outcome for this systematic review was to assess the evidence for an influence of breathing phase on pupil dynamics. Six studies included in this review measured effects of breathing phase [5, 18, 28, 43, 44, 47].

From the studies directly investigating whether breathing phase affects pupil dynamics, phase coherence between breathing and changes in pupil size was reported both at rest and while participants performed a visual oddball task. These findings were interpreted as suggestive of synchronization between breathing and pupil activity [43]. Similarly, in a separate study, pupil dilation amplitudes were shown to exhibit significant variation across the breathing cycle, and nine out of ten participants showed significant phase locking of breathing and pupil diameter. Additionally, in the same study, significant information transfer between breathing and pupil diameter was shown by multivariate granger causality [44]. Furthermore, it was found that pupil diameter fluctuated significantly across the breathing cycle before the test section of a match-to-sample version of a visual recognition task. However, during the task, there was only partial respiratory fluctuation in pupil size for the matching process, and no respiratory fluctuation in pupil size for the mismatching process. These results led the authors to conclude that respiratory fluctuations in pupil diameter are not maintained during cognitive tasks [47]. Overall, all three studies report findings that indicate that breathing phase influences pupil dynamics, however, it remains unclear whether this is driven by inhalation or exhalation and how these breathing phases differ in their influence on pupil dynamics.

Indirect findings supporting that breathing phase affects pupil dynamics comes from a study investigating the physiological characteristics of self-relatedness, using a thought-sampling task. Neither pupil diameter nor breathing phase covaried with self-relatedness, allowing for the possibility that they are affected in a similar manner [5].

The final two studies measuring breathing phase, investigated the effect of breathing phase on pupillary light reflex dynamics. Golenhofen and Petrányi (1967) reported that during spontaneous breathing, light stimulation during inhalation led to a significant increase in response latency and time to pupil redilation, as well as a decrease of pupil constriction amplitude and constriction time, compared to light stimulation during exhalation [18]. Similarly, Kaulen and colleagues (1979) divided the breathing cycle into four phases, early and late inhalation and exhalation, and reported significantly shorter pupillary response latency to light stimulation during the early inhalation phase compared to the late exhalation phase, as well as a shorter response latency during the early compared to the late exhalation phase. All other measured pupillary light reflex dynamics were not significantly affected by the breathing phase [28].

**Fig. 2:**
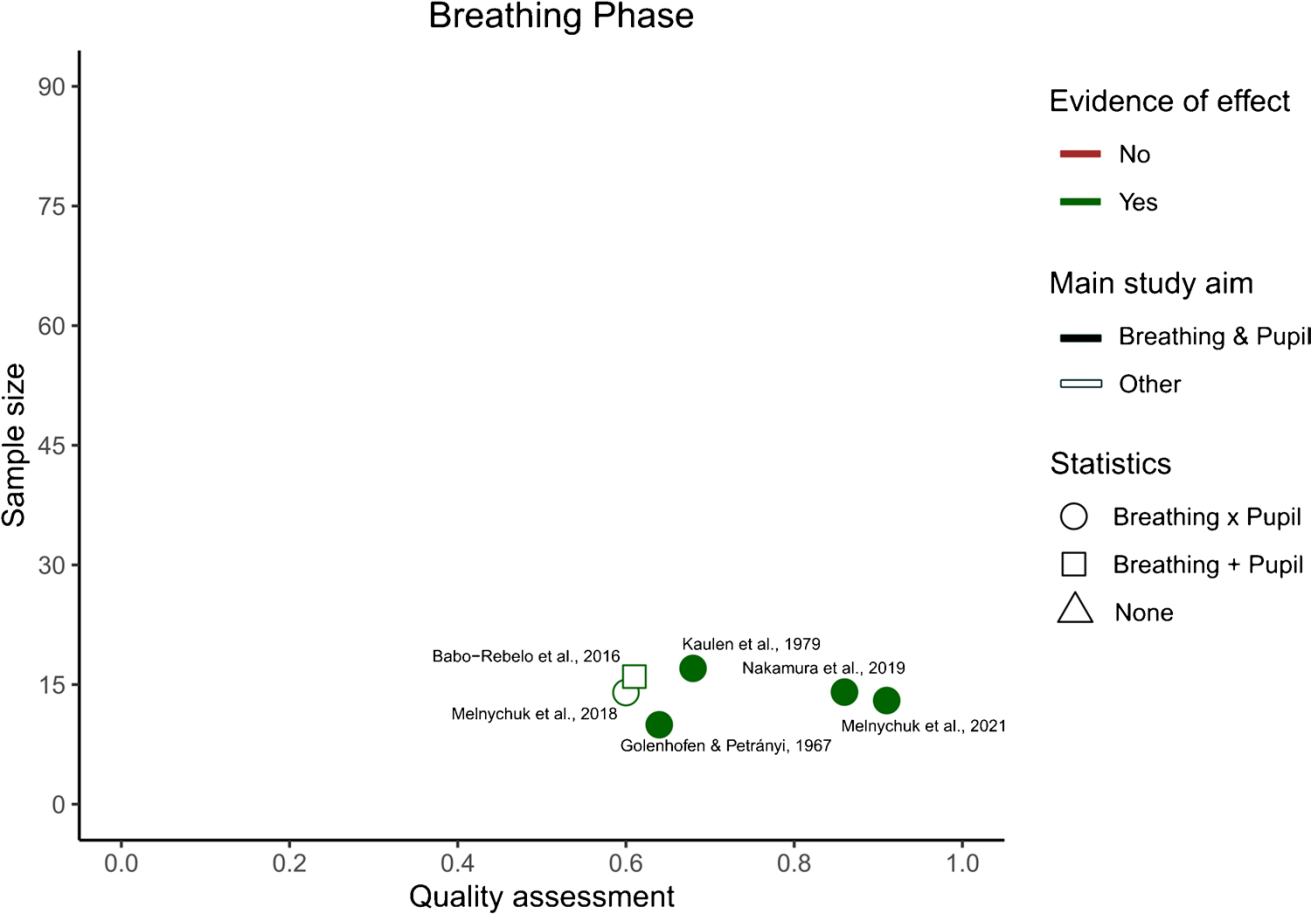
Breathing phase study overview. The x-axis depicts the quality assessment score each individual study received (1 being the highest and 0 the lowest possible score), and the *y*-axis depicts the number of participants included in each study for the breathing phase outcome. Each individual shape represents one study. The color of the shape indicates whether the study outcomes align with there being an effect of breathing phase on pupil dynamics. The shape fill indicates whether the main outcome of the study is breathing and pupil dynamics interactions or not. Finally, the form of the shape indicates whether the breathing and pupil related outcomes of the study have been analyzed statistically, and if so, whether changes in breathing phase and pupil dynamics were assessed separately ( +), or whether statistics were used to look at an interaction (x)

Taken together these results indicate that breathing phase might affect both pupillary light reflex dynamics, as well as fluctuations in pupil diameter at rest (Fig. 2).

#### Breathing depth

Six studies included in this review reported results for measures of breathing depth, volume, or amplitude [15, 21, 36, 50, 59, 65].

During normal spontaneous breathing, as well as when subjects were asked to voluntarily vary their breathing depth, the amplitude of pupil size fluctuations correlated highly with breathing tidal volume [50]. Furthermore, when participants switched from spontaneous breathing at rest, to slower (6 breaths per minute) deep breathing, their mean pupil diameter significantly increased. Additionally, a significant increase in pupillary unrest index, and a significant decrease in sample entropy, was found during the deep breathing condition [65]. However, in an investigation into the physiological responses to a series of autonomic function tests, which included a 7-min period of deep breathing at 6 bpm, no significant changes in pupil diameters were discovered [15].

Further, although of a more anecdotal character, indirect evidence comes from an individual that was able to voluntarily raise the hair on his body which led to an increase in pupil diameter and breathing depth [36].

Finally, measuring the physiological responses during a driving simulation task with various levels of difficulty revealed that breathing depth significantly decreases, whereas pupil diameter increases, as the driving task gets more difficult [21]. However, in another driving simulation study, only pupil size was found to significantly increase in response to increases in task difficulty without significant changes in breathing depth [59].

**Fig. 3:**
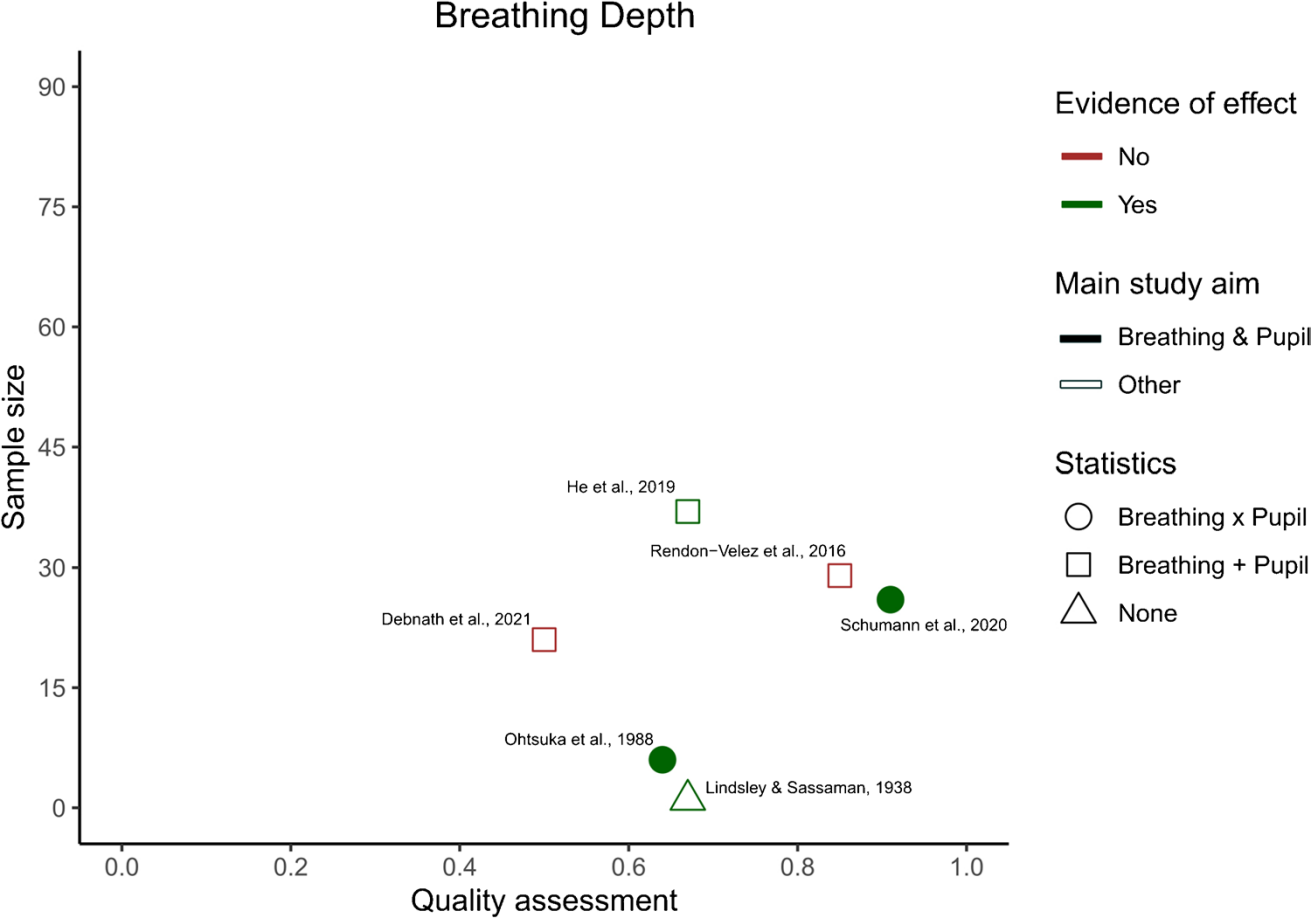
Breathing depth study overview. The *x*-axis depicts the quality assessment score each individual study received (1 being the highest and 0 the lowest possible score), and the *y*-axis depicts the number of participants included in each study for the breathing depth outcome. Each individual shape represents one study. The color of the shape indicates whether the study outcomes align with there being an effect of breathing depth on pupil dynamics. The shape fill indicates whether the main outcome of the study is breathing and pupil dynamics interactions or not. Finally, the form of the shape indicates whether the breathing and pupil related outcomes of the study have been analyzed statistically, and if so, whether changes in breathing depth and pupil dynamics were assessed separately ( +), or whether statistics were used to look at an interaction (*x*)

Taken together, these studies indicate that breathing depth might affect both pupil diameter and other pupil dynamics. However, as only two studies have addressed this topic directly, results remain inconclusive (Fig. 3).

#### Breathing rate

**Fig. 4:**
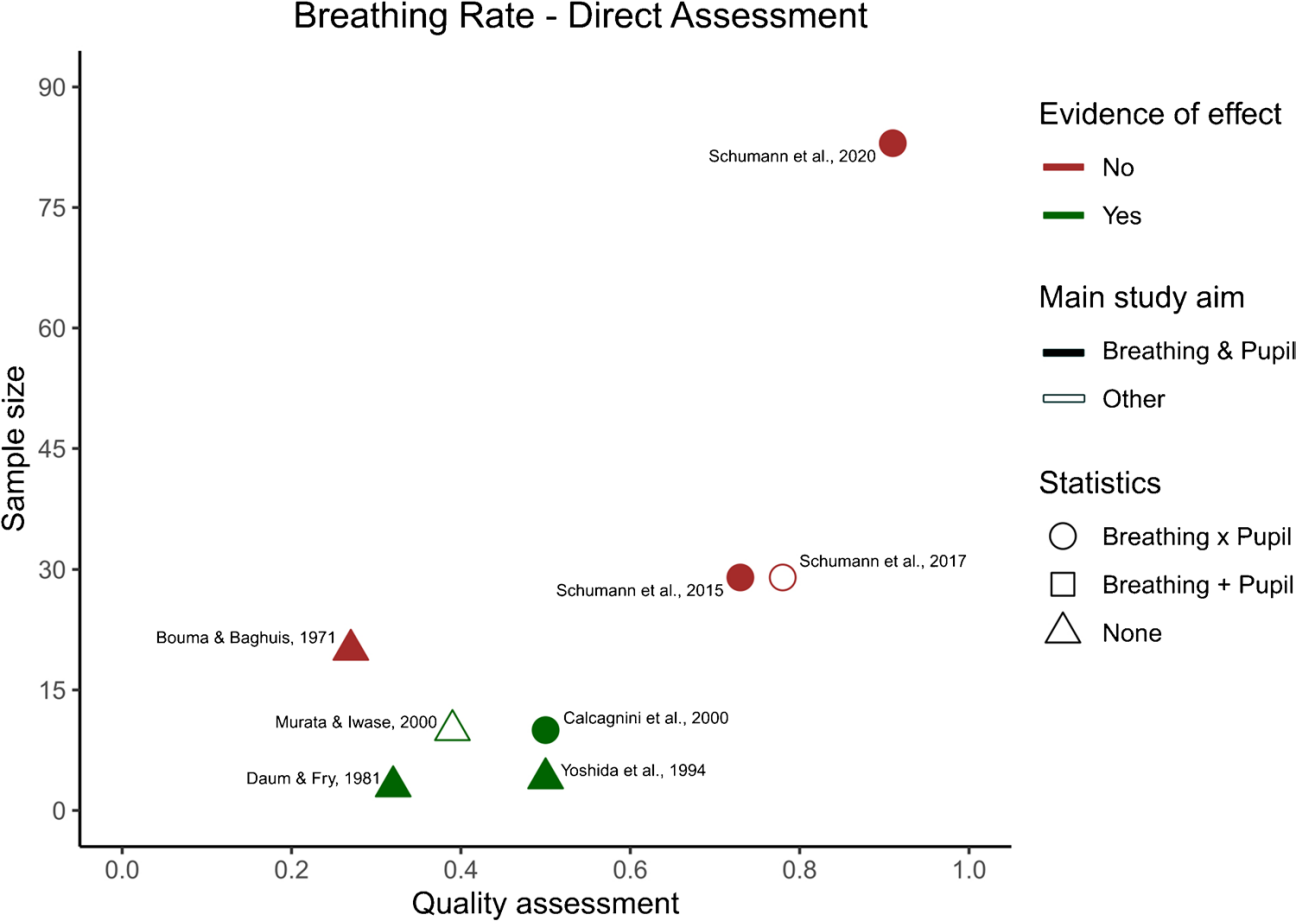
Breathing rate direct assessment study overview. The *x*-axis depicts the quality assessment score each individual study received (1 being the highest and 0 the lowest possible score), and the *y*-axis depicts the number of participants included in each study for the breathing rate outcome. Each individual shape represents one study. The color of the shape indicates whether the study outcomes align with there being an effect of breathing rate on pupil dynamics. The shape fill indicates whether the main outcome of the study is breathing and pupil dynamics interactions or not. Finally, the form of the shape indicates whether the breathing and pupil related outcomes of the study have been analyzed statistically, and if so, whether changes in breathing rate and pupil dynamics were assessed separately ( +), or whether statistics were used to look at an interaction (*x*)

**Fig. 5:**
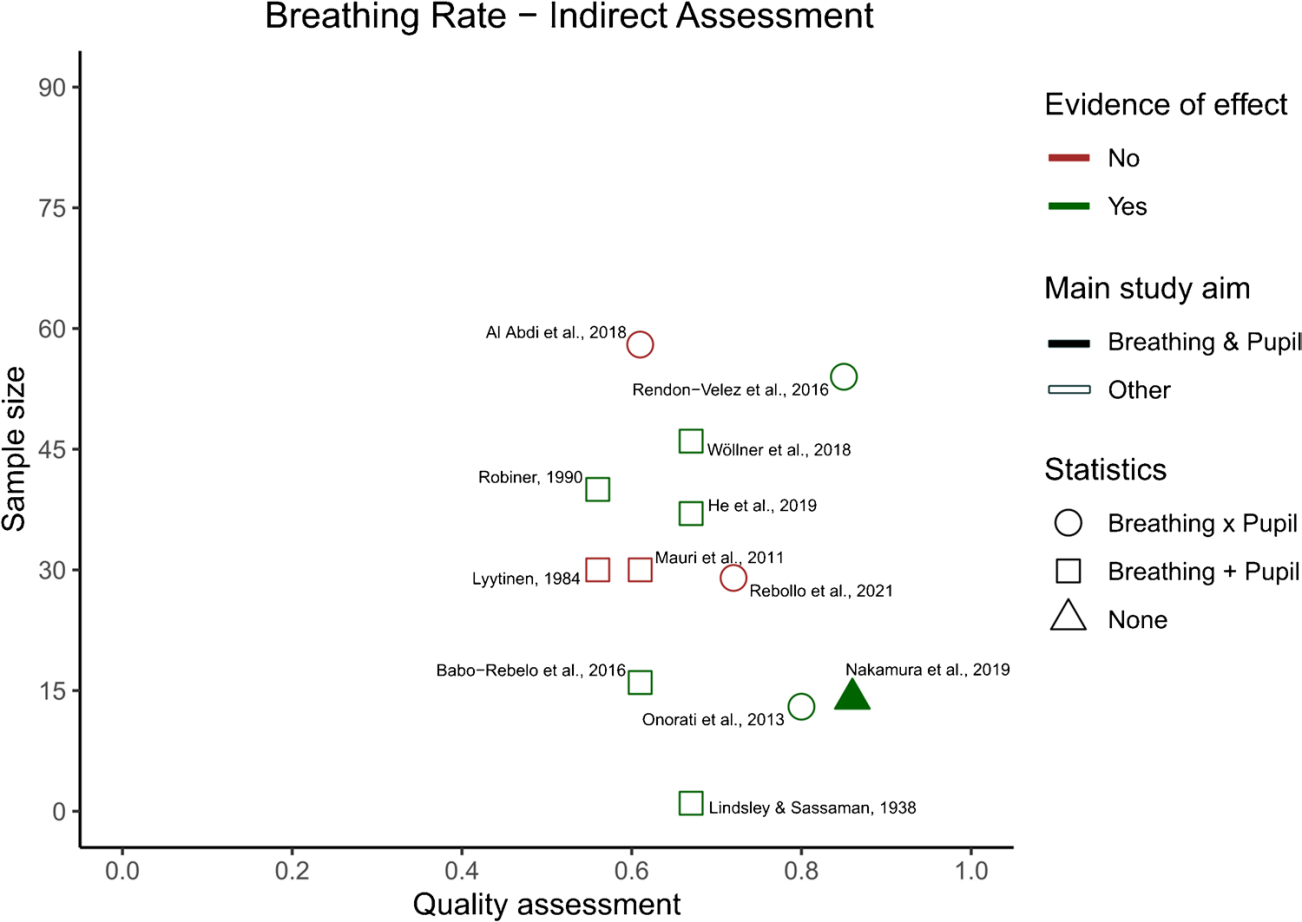
Breathing rate indirect assessment study overview. The *x*-axis depicts the quality assessment score each individual study received (1 being the highest and 0 the lowest possible score), and the *y*-axis depicts the number of participants included in each study for the breathing rate outcome. Each individual shape represents one study. The color of the shape indicates whether the study outcomes align with there being an effect of breathing rate on pupil dynamics. The shape fill indicates whether the main outcome of the study is breathing and pupil dynamics interactions or not. Finally, the form of the shape indicates whether the breathing and pupil related outcomes of the study have been analyzed statistically, and if so, whether changes in breathing rate and pupil dynamics were assessed separately ( +), or whether statistics were used to look at an interaction (*x*)

Breathing rate was an additional study outcome, and it was reported by 20 studies included in this systematic review (see Table 1). However, in most studies, it was only mentioned anecdotally. Therefore, we decided to divide the studies covering breathing rate into two groups; the studies that talked about the effect of breathing rate on pupil dynamics directly, and studies where this could be assessed indirectly. This was generally in line with the distinction of whether a study had breathing and pupil dynamics as part of their main study outcome, but there were three exceptions. Two studies did not have breathing and pupil dynamics as part of their main study outcome, but they reported results that were directly related to the question of whether breathing rate influences pupil dynamics (see Fig. 4) [46, 64]. On the other hand, even though Nakamura and colleagues (2019) focused on investigating the effect of breathing phase on pupil size, they also measured breathing rate. By assessing their results, we noticed that a significant increase in breathing rate was accompanied by a tendency for smaller fluctuations in pupil size during part of their task; however, this was not statistically investigated and therefore we classified this study as only indirectly assessing breathing rate (see Fig. 5) [47].

##### Direct measurements of pupil dynamics as a function of breathing rate

Calcagnini and colleagues (2001) assessed the effect of breathing on pupil function directly, demonstrating that participants breathing at a controlled rate of 15 breaths per minute showed a breathing component in the power spectrum of the pupilgram. Additionally, there was significant coherence between breathing and pupil size at the 0.25 Hz (the breathing rate) frequency band [11]. This is in line with previous findings of a breathing component in the power spectrum of the pupil diameter at the frequency of breathing [75]. These results are also supported by Murata and Iwase (2000) who studied pupil dynamics as a proxy for mental workload. Their results showed a peak in the power spectral density of the pupil size around the breathing frequency; however, this peak disappeared as the task difficulty increased [46]. Furthermore, the frequencies of different breathing rates were reflected in the frequency of pupil fluctuations, as shown by a partial correlation between the two [14].

In contrast, several studies with a considerably larger sample size, found no significant correlations between breathing rate and pupil diameter or pupillary unrest indices [64–66]. Moreover, Bouma and Baghuis (1971) investigated pupillary hippus (i.e., normal rhythmic fluctuations of the pupil) and showed that this was not related to breathing rate [9].

A summary of these studies can be seen in Fig. 4.

##### Indirect measurements of pupil dynamics as a function of breathing rate

Eight studies show indirect evidence for a potential correlation between breathing rate and pupil dynamics [5, 21, 36, 47, 51, 59, 60, 74].

For example, Nakamura and colleagues (2019) showed that a significant increase in breathing rate was accompanied by a tendency for smaller fluctuations in pupil size during the test section of a match-to-sample task, compared to the delay section of the task. However, this was not statistically investigated [47]. Similarly, in a study trying to characterize affective states with physiological correlates, significant coherence between breathing rate and pupil size was found in the majority of the participants during both the baseline and the affective states (happiness, anger, sadness) [51]. Furthermore, watching videos in slow motion or with music led to a change in both pupil diameter and breathing rate, where the former led to a decrease, and the latter to an increase, compared to watching the original video (normal speed, no music). However, movie genre affected only pupil sizes. [74]. In a study using a driving simulation paradigm, participants’ pupil diameter and breathing rate increased significantly when put under time pressure [59]. In a similar study, increasing the cognitive load during a driving simulation task led to increases in pupil diameter and breathing rate [21]. Moreover, in a case study on voluntary hair raising, as well as changes in breathing depth (as already mentioned), they observed an increase in pupil diameter and breathing rate [36].

Further indirect evidence for a potential link between breathing rate and pupil dynamics comes from two studies which report coinciding absences of effects on breathing and pupil parameters. One study found no covariation between self-relatedness (based on a thought-sampling task) and pupil diameter or breathing rate [5]. Likewise, immersion into whirlpools or warm tubs did not lead to significant changes in pupil size or breathing rate of the participants [60].

Four studies present indirect evidence against a potential correlation between breathing rate and pupil dynamics, because breathing rate and pupil dynamics differed in whether they were affected by a certain stimulus [1, 39, 42, 58].

No correlation between breathing rate and pupil size was found when presenting various types of food stimuli in fed or fasted states [58]. Similarly, in response to arithmetic-induced stress, pupil diameter increased with cognitive load, but breathing remained unchanged [1]. Furthermore, in a study on task switching, while pupil diameter and breathing rate decreased when switching to using Facebook from a relaxing scenario, switching further to mental tasks led to reduced breathing but increased pupil size. [42]. Finally, exposing participants to a wide variety of stimuli, ranging from electrical shocks to arithmetic tasks, led to inconsistent and asynchronous effects on breathing rate and pupil size [39].

For a summary of these results, see Fig. 5.

Finally, three additional studies measured breathing rate and pupil dynamics; however, the nature of their results makes it difficult to determine whether they lend support for or against the theory that breathing rate influences pupil dynamics.

A sham vagus nerve stimulation of the left earlobe during a predictive learning task did not lead to significant changes in breathing rate or pupil size. However, this held true only when excluding participants for which the procedure was performed in a suboptimal manner, including these participants in the analysis led to a significant increase in pupil size, making interpretation hard [12]. Further ambiguous results came from a study that found that breathing and pupillary responses to images varies depending on group (control or spider phobic) and image content (butterflies, birds or spiders). However, a correlation between breathing rate and pupil size was not assessed [4]. Finally, an investigation into the effects of slow breathing on the pupillary light reflex found indications of a slight increase in redilation time; however, these effects were smaller than the inter-individual variations and they were not tested statistically, making it impossible to classify this study [18].

#### Additional breathing measures

Besides breathing phase, depth, and rate we had preregistered breathing route (nose/mouth breathing) as a parameter of interest, but no studies included in this systematic review investigated breathing route. However, four included studies reported breathing measures that did not clearly fall under the predefined parameters of breathing phase, depth, rate, or route.

Two studies reported a combination of breathing rate and depth as their breathing measure. One of these measures was respiration line length, which was recorded simultaneously with pupil size while participants performed a relevant comparison test where they were either guilty or innocent of committing a mock crime. The participants that were asked to be deceptive about their mock crime during “interrogation” had increased pupil sizes and decreased breathing [24]. Furthermore, an autoregressive model that was developed to predict variations in pupil size, used the breathing signal as input and could thereby significantly account for around 3% of the variance [77].

Gavriysky and colleagues (1991) investigated the effect of hyperventilation (20 breaths/min) on pupillary light reflex dynamics and found a significant increase in response latency during hyperventilation, a significant increase in pupil constriction time during the first and after the second hyperventilation period, and a tendency for the pupil constriction amplitude to decrease during hyperventilation [17].

Finally, Lyytinen (1984) investigated respiratory disturbances in the form of nonstandard respiratory cycles, however this did not significantly change throughout his study, despite a variety of stimuli ranging from electric shocks to cognitive tasks. Pupil size, on the other hand, reliably constricted and then dilated in response to the cued pre-stimulus period. However, pupil size was only measured in a subset of participants across a wide range of stimuli and time periods, leading to various effects on pupil size that were difficult to summarize [39].

### Certainty of evidence

The certainty of evidence was evaluated with the GRADE method for the three main parameters; how breathing phase, depth, and rate affect pupil dynamics [20]. The final GRADE score is shown in Table 3. This evaluation led us to conclude that the certainty of evidence that breathing phase affects pupil dynamics is “low”. The evidence that breathing depth or breathing rate affect pupil dynamics was estimated as “very low”.

## Discussion

We set out to review and synthesize the literature regarding the fundamental question of how breathing influences pupil dynamics in humans. Our main aim was to capture the effects of breathing phase, with the additional aims of describing results for breathing depth, rate and route. Based on state-of-the-art reviews [3, 48] targeting the relationship between breathing and higher functions, it is easy to get the impression that the theoretical and factual link between breathing and pupil dynamics is established and well-studied. However, our systematic review, which summarizes research from more than 30 articles, spanning over 80 years of inquiry, tells a different story. We find that since Golenhofen and Petrányi’s (1967) study, the first to explicitly test this question in humans, only 11 studies have been conducted that asses this relationship directly. Below we will address the current evidence for the theory that breathing can influence pupil dynamics for each breathing parameter separately, and the limitations and outstanding questions that need to be solved before a conclusive answer can be given of its validity.

### Phase

Six of the included studies looked at breathing phase, and among these studies there was a diversity of study methods and aims; however, all of them aligned with the hypothesis that breathing phase influences pupil dynamics. When setting out to conduct this systematic literature review, we expected to find how inhalation and exhalation specifically affect pupil function. However, focus in the reviewed literature was instead on changes in pupil dynamics over the course of the breathing cycle in general. Therefore, we can only say that from the studies included in this systematic review, breathing phase appears to correlate with pupil size and pupil size fluctuations.

Generally, these studies have small sample sizes which reduces the strength of their findings. Furthermore, the wide variety of study methods and outcome measures made it difficult to summarize their findings. The final GRADE score for this category rated the strength of evidence as “low”, and with that the evidence for an effect of breathing phase on pupil dynamics is not negligible but far from conclusive.

### Depth

The included studies assessing breathing depth were not as homogenous in their results as those assessing phase, with only four out of six studies finding evidence for the theory that breathing depth affects pupil dynamics. Of the four studies that found an effect of breathing depth on pupil dynamics, three studies reported that an increase in breathing depth also led to an increase in pupil size, and one study even reported a linear correlation between breathing depth and pupil size [50]. However, since He and colleagues (2019) reported the opposite effect, and two studies did not find an effect at all, it is not clear if, and how, breathing depth influences pupil dynamics [15, 21, 59]. In addition, the majority of these studies did not set out to investigate the effect of breathing depth on pupil dynamics and further suffered from statistical shortcomings. This led to the quality of the evidence surrounding effects of breathing depth on pupil dynamics to be classified as “very low” according to the GRADE criteria. Therefore, even though the majority of studies that measure breathing depth and pupil dynamics are in favor of a correlation between these two measures, until further research is published, we are hesitant to place much confidence in the existence of such an effect.

### Rate

Breathing rate was the most common breathing measure reported in the studies included in this systematic review (20 out of 31). Because we had such a large number of relevant studies, we decided to split them into two categories; the studies that reported on effects of breathing rate on pupil dynamics directly, and those that did so only indirectly. We made these classifications based on the main study outcome and the reported results of the included studies.

Of the eight studies classified as providing direct evidence on the question of whether breathing rate influences pupil dynamics, four studies reported results in favor of an effect, whereas the other four studies did not (see Fig. 4).

Importantly, while the absence of evidence in these studies should not be taken as evidence of absence, the studies that did not find an effect were generally rated as being of higher quality, and had larger sample sizes than those that found an effect. This discrepancy between the presence of an effect in studies with a smaller sample size and the absence of the effect in studies with larger sample size might point towards a more widespread lack of statistical power and potential false rejections of the null hypothesis in some of the studies included in this review [6, 10, 62]. Specifically, small sample sizes, like the ones observed in the majority of the included studies, are only appropriate if the effects being estimated are very large which is unlikely in this case [10].

The studies that indirectly reported breathing rate and pupil dynamics measures are larger in quantity [12], and on average have a larger sample size and better rated study quality for their intended study aim, than the studies that directly investigate breathing rate and pupil dynamics (see Fig. 5). The majority of these indirect studies (8 out of 12) lend support to the hypothesis that breathing rate influences pupil dynamics. However, due to the indirect nature of these studies and the inconsistency of their findings, these studies still received an overall GRADE score of “very low”. In addition, the majority of the studies giving indirect support to the theory that breathing rate influences pupil dynamics, did so by reporting a simultaneous increase in breathing rate and pupil size. In contrast, the studies that found an effect when directly investigating breathing rate and pupil size, mainly reported the presence of a peak in the power spectrum of the pupil size at the breathing frequency. This difference in the kind of measured outcomes makes it hard to integrate both kinds of findings. However, despite the weakness of the evidence in favor of an effect of breathing rate on pupil dynamics, the relatively large number of studies finding some sort of covariance between these two measures cautions us against discarding this hypothesis altogether.

### Pupil parameters

For this systematic review, we grouped studies according to the breathing parameters, given that our main aim was the effects of breathing on pupil dynamics. However, it is also possible to do the reverse, and group the study results by type of pupil measurements, which could contribute with more pupil-specific information. Specifically, the pupillary outcomes included in this review are: pupil size, pupillary unrest (hippus), pupil size fluctuations, and pupillary light reflex dynamics.

Shifting to a pupil-centered perspective, out of the five studies that discussed pupillary unrest indices or hippus (see Tables 1 and 2), only one study reported an effect of breathing (breathing rate) [14]. However, it should be noted that they did discuss pupillary unrest more or less interchangeably with pupil size and pupil size fluctuations. The other four studies measuring breathing rate did not find an effect, and one study also looked at breathing depth on pupillary unrest and found no effect [65]. Among studies measuring pupillary unrest, there was variation in how they measured this, as well as their assessed quality; however, since this was not one of our main study outcomes, we did not formally assess the quality of this evidence.

All four studies reporting pupil size fluctuations found that these were influenced by either breathing rate or breathing depth [11, 14, 50, 75]. However, again, there was an overlap with both pupillary unrest and pupil size in their measurements, but it is unclear how much pupillary unrest and pupil size fluctuations are distinct from pupil size in these cases.

Finally, three of the articles measured the effect of various aspects of breathing on the response of the pupil to sudden light stimulation, which we categorized as pupillary light reflex dynamics [17, 18, 28]. Together they looked at breathing phase, rate, and the effect of hyperventilation, and all reported some effect on pupillary light reflex dynamics. However, these studies were inconsistent in their findings, and all suffer from various methodological shortcomings (e.g., small sample sizes), thereby weakening the strength of their findings. Note, though, that, as above, we did not formally assess the strength of evidence for this outcome.

### Influence of experimental paradigms

A wide variety of studies have been included in this review, some that support the theory that breathing influences pupil dynamics, and some that do not. Importantly, whether or not these studies measured breathing and pupil at rest, or employed a certain set of tasks or stimuli, does not seem to be a good predictor of whether indications of breathing influences on pupil dynamics will be found. However, a few studies reported a change in both breathing and pupil measures, and sometimes a change in their interaction, as a result of increases in cognitive load or task difficulty [21, 46, 47, 59]. Two studies report significant increases in both pupil size and breathing rate in response to increasing cognitive demands during driving simulation tests [21, 59]. On the other hand, two other studies found that the interaction between breathing rate and pupil size disappeared when the participants task demands increased [46, 47]. However, there were several other studies that included cognitive tasks of various difficulty into their study design and did not report significant simultaneous effects on breathing and pupil measures. A firm conclusion cannot be made based on these results, but it is salient to highlight that potential interactions with study paradigms warrants further investigation.

### Limitations of the review processes

This review follows the PRISMA guidelines and the assessment of the certainty of the evidence was based on the GRADE approach. As both these methods have been created with clinical trials in mind, we modified some of the steps of these methods and excluded others. The included steps on the PRISMA guidelines can be seen in Table 1 in the Supplementary materials while the utilization of GRADE is discussed in the “Methods” section with a more detailed discussion in the “Detailed GRADE assessment” section of the Supplementary materials.

Our modifications to the GRADE approach were influenced by the large variability in study design due to the broad inclusion criteria, and the lack of statistical assessment of the relationship between breathing and pupil dynamics. This made the GRADE process somewhat more subjective and imprecise, such as the initial GRADE scoring of the study design, and the assessment of the imprecision of the reported results. We did not assess publication bias. However, publication bias is another potentially very large source of bias. Importantly, because inclusion of the “publication bias” criterion would only have reduced the GRADE score of any outcome measure, we do not see it as likely to change the results of the GRADE, which were all “low” or “very low”.

Moreover, as we did not do a meta-analysis and perform any statistical analysis of the body of evidence, this could potentially bias the findings by making it harder to give appropriate weighting to low-quality studies with low sample sizes. However, the quality assessment of the individual articles, and the GRADE approach to assess the overall evidence for each parameter, were designed to take this bias into account.

While the experimental setup and processing of the data was evaluated through the quality assessment, it was beyond the scope of this review to assess this thoroughly. For example, we did not assess how the different software used for pupil measurements, estimated pupil size. However, because some methods to assess pupil diameter are more reliable than others, looking at the measurement setup and analysis in more detail could provide a more accurate account of the reliability of the study outcomes [13, 35].

### Limitations of included studies and implications for future research

During the last few years, the potential impact of breathing on behavior and brain function has received a flurry of attention. It is therefore critical to understand the extent of the interactions between breathing and pupil dynamics which has often been taken as a given. On the face of it, the question of whether breathing influences pupil size seems relatively simple and straightforward to answer. What we show in this systematic review is that this does not seem to be the case. There is no conclusive evidence that breathing phase, depth, or rate influences pupil dynamics. Surprisingly, despite research spanning many decades the fundamental question of whether breathing route impacts pupil function has, to the best of our knowledge not yet been investigated.

To clearly answer if there is a connection between breathing and pupil function, it is necessary to conduct research with a higher quality than before and a direct focus on this relationship. We believe that pre-registered studies using modern equipment, adequate sample sizes, proper statistics, and a lack of confounding stimuli should be able to answer the fundamentals of this question with relative ease. In Box 1, we outline a series of outstanding questions and potential confounds that future studies should address. Until such research has been carried out, we caution against strong trust in an effect of breathing on pupil dynamics. For example, the majority of the studies in this review measure breathing with a breathing belt. This is unfortunate as breathing belts have both poor reliability and validity [25]. Moreover, they do not separate nose from mouth breathing, which is of critical importance as the underlying central mechanisms and behavioral outcome between these two formats of breathing are very different [2, 22, 27, 69]. We also noticed that many studies do not measure or take account for eye blinks. This is critical because breathing affects the probability of eye blinks, which in turn affects pupil size. For example, blinking is more likely during exhalation [63] which means that the probability that inhalation and pupil dilation (when you open your eyes) will coincide is larger compared to exhalation. Likewise, the potential of cardio-respiratory coupling [55] also stresses the importance of controlling for heart rate, which is known to influence pupil size [71].

Box 1 Questions for future research and possible confounds

**Table:** 

**Questions for future research** • Is there a differential effect of breathing route: nose vs mouth breathing? • What neural pathways are involved? • Does attention modulate the effect? • Does arousal modulate the effect? • How sensitive is the effect to external stimuli and tasks? • Is there a sex difference in the effect?	**Possible confounds** **Breathing** • Breathing measurements [25] **Pupil** • Eye blinks [13] • Lighting conditions [72] • Measurements from both eyes or only one [56] • Differences in the algorithms and methods used to extract the pupil data [35] **Other** • Cardiac activity [65] • Arousal [71] • Cognitive load [26] • Attention [70]

## Conclusion

Given the fundamental nature and importance of the connection between the central mechanism of breathing and pupil function, we urge the field to carefully address the many outstanding questions that remain. While this systematic review concerned humans, we believe that similar uncertainties may be present for non-human animal studies. In fact, although we have not done a formal systematic review, we suspect that there may be even fewer studies that have addressed this question directly in animal models. Taken together, this old question deserves new inquiries which we believe have the power to not only give a final answer, but to go on to inform new research questions.

## Supplementary Information

Below is the link to the electronic supplementary material.

Supplementary file1 (DOCX 692 kb)

## Data Availability

Data sharing not applicable to this article as no datasets were generated or analyzed during the current study.
