# Supplementary material for: Inconclusive evidence that breathing shapes pupil dynamics in humans: a systematic review
Source: Pflugers Arch. 2022 Jul 25;475(1):119–37. doi: 10.1007/s00424-022-02729-0 (PMC9816272; doi:10.1007/s00424-022-02729-0)
Supplement: Supplementary file 1 — Supplementary file1 (DOCX 692 kb) [file 424_2022_2729_MOESM1_ESM.docx]

# Supplemental material

| **Section and Topic** | **Item #** | **Checklist item** | **Reported on page #** |
| --- | --- | --- | --- |
| **TITLE** | | |  |
| Title | 1 | Identify the report as a systematic review. | 1 |
| **ABSTRACT** | | |  |
| Abstract | 2 | See the PRISMA 2020 for Abstracts checklist. | 2 |
| **INTRODUCTION** | | |  |
| Rationale | 3 | Describe the rationale for the review in the context of existing knowledge. | 3 |
| Objectives | 4 | Provide an explicit statement of the objective(s) or question(s) the review addresses. | 4 |
| **METHODS** | | |  |
| Eligibility criteria | 5 | Specify the inclusion and exclusion criteria for the review and how studies were grouped for the syntheses. | 5 |
| Information sources | 6 | Specify all databases, registers, websites, organizations, reference lists and other sources searched or consulted to identify studies. Specify the date when each source was last searched or consulted. | 5 |
| Search strategy | 7 | Present the full search strategies for all databases, registers and websites, including any filters and limits used. | 5, 42-43 |
| Selection process | 8 | Specify the methods used to decide whether a study met the inclusion criteria of the review, including how many reviewers screened each record and each report retrieved, whether they worked independently, and if applicable, details of automation tools used in the process. | 5-6 |
| Data collection process | 9 | Specify the methods used to collect data from reports, including how many reviewers collected data from each report, whether they worked independently, any processes for obtaining or confirming data from study investigators, and if applicable, details of automation tools used in the process. | 5-6 |
| Data items | 10a | List and define all outcomes for which data were sought. Specify whether all results that were compatible with each outcome domain in each study were sought (e.g. for all measures, time points, analyses), and if not, the methods used to decide which results to collect. | 4 |
|  | 10b | List and define all other variables for which data were sought (e.g. participant and intervention characteristics, funding sources). Describe any assumptions made about any missing or unclear information. | NA |
| Study risk of bias assessment | 11 | Specify the methods used to assess risk of bias in the included studies, including details of the tool(s) used, how many reviewers assessed each study and whether they worked independently, and if applicable, details of automation tools used in the process. | 6-7 |
| Effect measures | 12 | Specify for each outcome the effect measure(s) (e.g. risk ratio, mean difference) used in the synthesis or presentation of results. | NA |
| Synthesis methods | 13a | Describe the processes used to decide which studies were eligible for each synthesis (e.g. tabulating the study intervention characteristics and comparing against the planned groups for each synthesis (item #5)). | 7 |
|  | 13b | Describe any methods required to prepare the data for presentation or synthesis, such as handling of missing summary statistics, or data conversions. | NA |
|  | 13c | Describe any methods used to tabulate or visually display results of individual studies and syntheses. | 7 |
|  | 13d | Describe any methods used to synthesize results and provide a rationale for the choice(s). If meta-analysis was performed, describe the model(s), method(s) to identify the presence and extent of statistical heterogeneity, and software package(s) used. | 7 |
|  | 13e | Describe any methods used to explore possible causes of heterogeneity among study results (e.g. subgroup analysis, meta-regression). | NA |
|  | 13f | Describe any sensitivity analyses conducted to assess robustness of the synthesized results. | NA |
| Reporting bias assessment | 14 | Describe any methods used to assess risk of bias due to missing results in a synthesis (arising from reporting biases). | NA |
| Certainty assessment | 15 | Describe any methods used to assess certainty (or confidence) in the body of evidence for an outcome. | 8-9, 47-48 |
| **RESULTS** | | |  |
| Study selection | 16a | Describe the results of the search and selection process, from the number of records identified in the search to the number of studies included in the review, ideally using a flow diagram. | 9 |
|  | 16b | Cite studies that might appear to meet the inclusion criteria, but which were excluded, and explain why they were excluded. | 9 |
| Study characteristics | 17 | Cite each included study and present its characteristics. | 11-16 |
| Risk of bias in studies | 18 | Present assessments of risk of bias for each included study. | 45-46 |
| Results of individual studies | 19 | For all outcomes, present, for each study: (a) summary statistics for each group (where appropriate) and (b) an effect estimate and its precision (e.g. confidence/credible interval), ideally using structured tables or plots. | 16-24 |
| Results of syntheses | 20a | For each synthesis, briefly summarize the characteristics and risk of bias among contributing studies. | 16-24 |
|  | 20b | Present results of all statistical syntheses conducted. If meta-analysis was done, present for each the summary estimate and its precision (e.g. confidence/credible interval) and measures of statistical heterogeneity. If comparing groups, describe the direction of the effect. | NA |
|  | 20c | Present results of all investigations of possible causes of heterogeneity among study results. | NA |
|  | 20d | Present results of all sensitivity analyses conducted to assess the robustness of the synthesized results. | NA |
| Reporting biases | 21 | Present assessments of risk of bias due to missing results (arising from reporting biases) for each synthesis assessed. | NA |
| Certainty of evidence | 22 | Present assessments of certainty (or confidence) in the body of evidence for each outcome assessed. | 25 |
| **DISCUSSION** | | |  |
| Discussion | 23a | Provide a general interpretation of the results in the context of other evidence. | 26 |
|  | 23b | Discuss any limitations of the evidence included in the review. | 30 |
|  | 23c | Discuss any limitations of the review processes used. | 29 |
|  | 23d | Discuss implications of the results for practice, policy, and future research. | 30-31 |
| **OTHER INFORMATION** | | |  |
| Registration and protocol | 24a | Provide registration information for the review, including register name and registration number, or state that the review was not registered. | 1 |
|  | 24b | Indicate where the review protocol can be accessed, or state that a protocol was not prepared. | 1 |
|  | 24c | Describe and explain any amendments to information provided at registration or in the protocol. | NA |
| Support | 25 | Describe sources of financial or non-financial support for the review, and the role of the funders or sponsors in the review. | 1 |
| Competing interests | 26 | Declare any competing interests of review authors. | 1 |
| Availability of data, code and other materials | 27 | Report which of the following are publicly available and where they can be found: template data collection forms; data extracted from included studies; data used for all analyses; analytic code; any other materials used in the review. | NA |

**Supplementary Table 1** – PRISMA checklist.

**Search strategy**

| **Medline** | |
| --- | --- |
| \| Interface: Ovid MEDLINE(R) and Epub  Ahead of Print, In-Process & Other Non-Indexed Citations and Daily  Date of Search: November 1, 2021  Number of hits: 1126  Comment: In Ovid, two or more words are automatically searched as phrases; i.e. no quotation marks are needed \| \| --- \| | \| Field labels   - exp/ = exploded MeSH term - / = non exploded MeSH term - .ti,ab,kf. = title, abstract and author keywords - adjx = within x words, regardless of order - = truncation of word for alternate endings \| \| --- \| |
| \| Database(s): **Ovid MEDLINE(R) and Epub Ahead of Print, In-Process, In-Data-Review & Other Non-Indexed Citations and Daily** 1946 to October 29, 2021  Search Strategy:   \| # \| Searches \| Results \| \| --- \| --- \| --- \| \| 1 \| Exp Pupil/ph [Physiology] \| 3483 \| \| 2 \| (pupil* or hippus).ab,kf,ti. \| 31730 \| \| 3 \| 1 or 2 \| 32280 \| \| 4 \| Exp Respiration/ \| 119187 \| \| 5 \| (respir* or inhal* or exhale* or breath*).ab,kf,ti. \| 763691 \| \| 6 \| 4 or 5 \| 804679 \| \| 7 \| 3 and 6 \| 1126 \| \| \| --- \| --- \| --- \| --- \| --- \| --- \| --- \| --- \| --- \| --- \| --- \| --- \| --- \| --- \| --- \| --- \| --- \| --- \| --- \| --- \| --- \| --- \| --- \| --- \| --- \| | |

**Supplementary Table 2** – Search strategy for the Medline database.

| **Web of Science Core Collection** | |
| --- | --- |
| \| Interface: Clarivate Analytics  Date of Search: November 1, 2021  Number of hits: 849 \| \| --- \| | Field labels   - TS/Topic = title, abstract, author keywords and Keywords Plus - NEAR/x = within x words, regardless of order - = truncation of word for alternate endings   Note: sometimes “quotation marks” are needed for single search terms to avoid automatic term mapping (lemmatization). |
| \| \| # \| Searches \| Results \| \| --- \| --- \| --- \| \| 1 \| TS=(pupil* or hippus) \| 42262 \| \| 2 \| TS=(respir* or inhal* or exhale* or breath*) \| 836438 \| \| 3 \| 1 AND 2 \| 849 \| \| \| --- \| --- \| --- \| --- \| --- \| --- \| --- \| --- \| --- \| --- \| --- \| --- \| --- \| | |

**Supplementary Table 3** – Search strategy for the Web of Science database.

| **PsycInfo** | |
| --- | --- |
| \| Interface: Ovid  Date of Search: November 1, 2021  Number of hits: 222  Comment: In Ovid, two or more words are automatically searched as phrases; i.e. no quotation marks are needed \| \| --- \| | Field labels   - exp/ = exploded controlled term - / = non exploded controlled term - .ti,ab,id. = title, abstract and author keywords - adjx = within x words, regardless of order - = truncation of word for alternate endings |
| \| \| # \| Searches \| Results \| \| --- \| --- \| --- \| \| 1 \| (pupil* or hippus).ab,hw,ti. \| 27401 \| \| 2 \| (respir* or inhal* or exhale* or breath*).ab,hw,ti. \| 39145 \| \| 3 \| 1 AND 2 \| 222 \| \| \| --- \| --- \| --- \| --- \| --- \| --- \| --- \| --- \| --- \| --- \| --- \| --- \| --- \| | |

**Supplementary Table 4** – Search strategy for the PsycInfo database.

| **Criteria** | | **Yes (2)** | **Partial (1)** | **No (0)** | **NA** |
| --- | --- | --- | --- | --- | --- |
| 1 | Question / objective sufficiently described? |  |  |  |  |
| 2 | Study design evident and appropriate? |  |  |  |  |
| 3 | Method of subject/comparison group selection or source of information/input variables described and appropriate? |  |  |  |  |
| 4 | Subject (and comparison group, if applicable) characteristics sufficiently described? |  |  |  |  |
| 5 | If interventional and random allocation was possible, was it described? |  |  |  |  |
| 6 | If interventional and blinding of investigators was possible, was it reported? |  |  |  |  |
| 7 | If interventional and blinding of subjects was possible, was it reported? |  |  |  |  |
| 8 | Outcome and (if applicable) exposure measure(s) well defined and robust to measurement / misclassification bias? Means of assessment reported? |  |  |  |  |
| 9 | Sample size appropriate? |  |  |  |  |
| 10 | Analytic methods described/justified and appropriate? |  |  |  |  |
| 11 | Some estimate of variance is reported for the main results? |  |  |  |  |
| 12 | Controlled for confounding? |  |  |  |  |
| 13 | Results reported in sufficient detail? |  |  |  |  |
| 14 | Conclusions supported by the results? |  |  |  |  |

**Supplementary Table 5** – Quality assessment questions from the QualSyst tool [34].


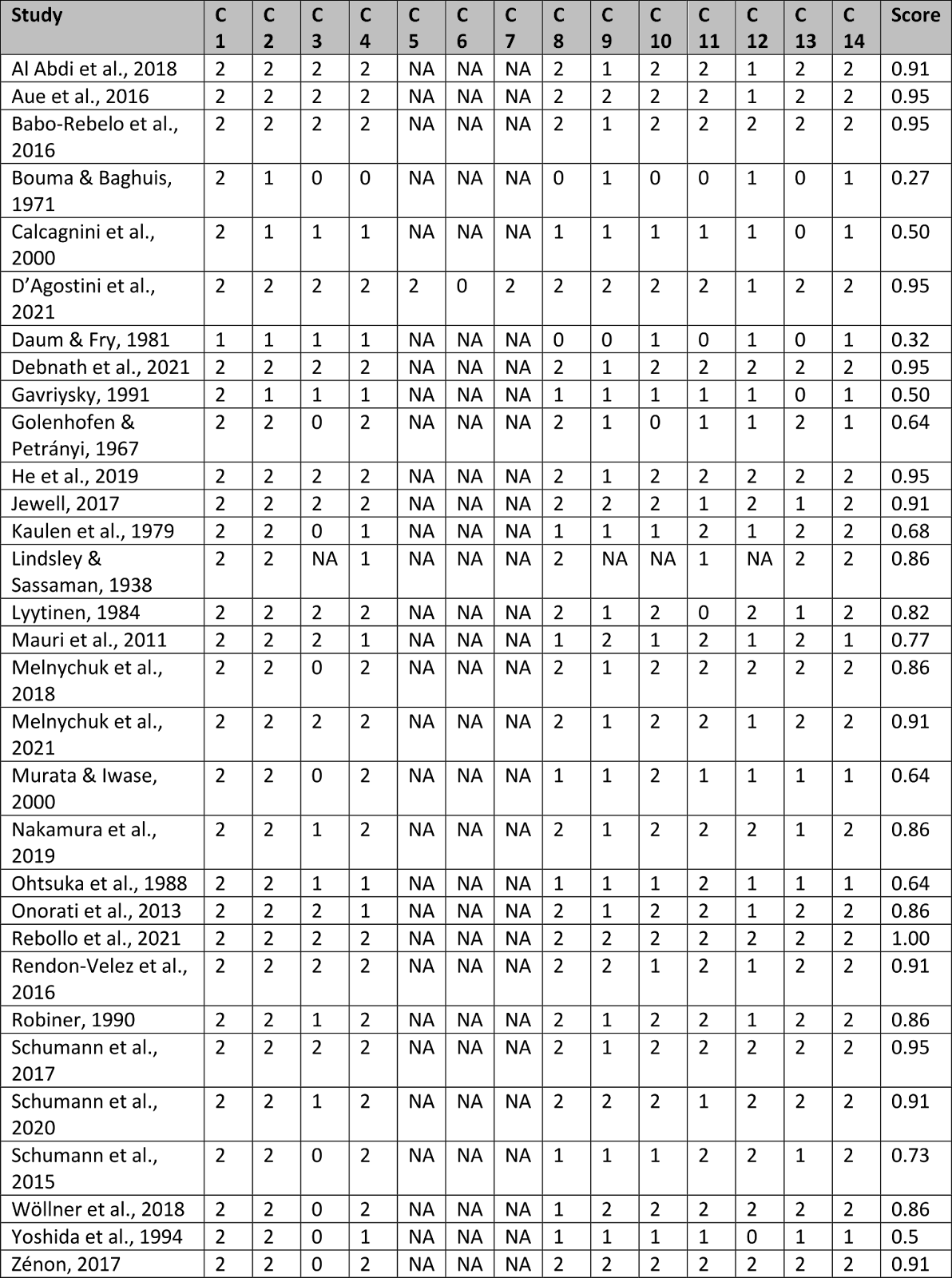


**Supplementary Table 6** – Quality assessment of all included studies. C1-C14 are the various quality assessment criteria (see Supplementary Table 5 for more detail). The criteria were rated as “NA” when that criterion was not applicable for the study design. The score column shows the final quality score for each study (the score can range from 0 to 1).


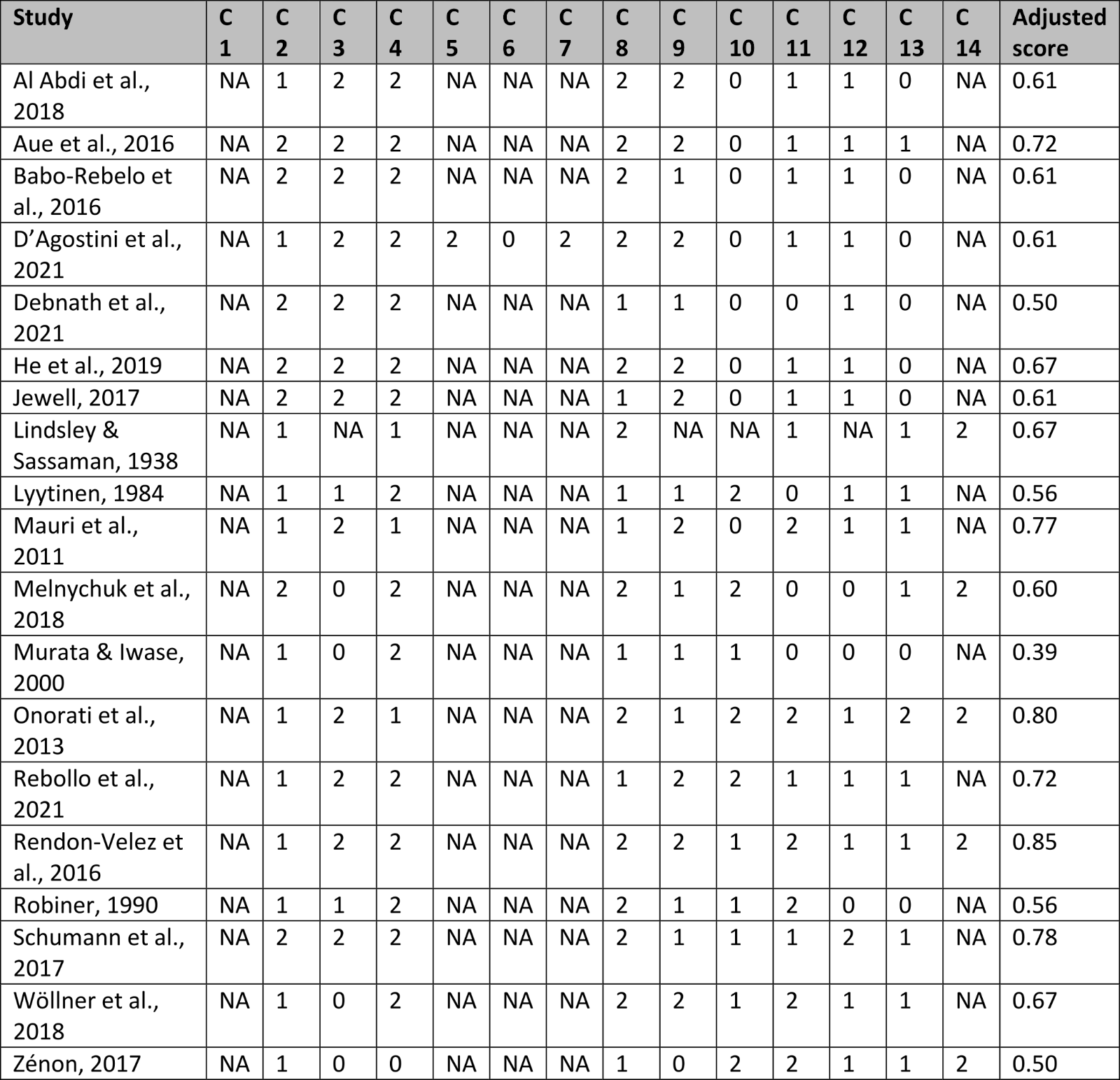


**Supplementary Table 7** – Adjusted quality assessment of included studies that did not have breathing and pupil size as their main study outcome. C1-C14 are the various quality assessment criteria (see Supplementary Table 5 for more detail). The criteria were rated as “NA” when that criterion was not applicable for the study design. The score column shows the final quality score for each study (the score can range from 0 to 1).

## Detailed GRADE assessment

For all outcomes, we gave more weight in the GRADE scoring process to those studies with larger sample sizes and those studies that more directly assessed the relationship of breathing and pupil dynamics. Very often the studies that were less direct were the ones that included a task, meaning the evidence is more certain for studies employing a resting-state design.

In assessing the initial GRADE level for study design, with the almost complete absence of RCTs, we used the various aspects of the quality assessment to guide us, where each author had a slightly different approach as to which aspects to take account of. For example, one author emphasized questions 2 and 3, which assessed the appropriateness of the study design and the appropriateness of the source of input variables, respectively, while another used the overall quality assessment score. Though this method was somewhat more subjective than the GRADE approach would ideally be, it was clear that for each outcome there were studies of sufficient quality to yield meaningful outcome, yet also not enough that were of very high quality. For this reason, we GRADEd the study design as moderate for all outcomes, a result which, crucially, all 3 authors came to independently.

Phase

Overall risk of bias was present because of the use of breathing belts as a measurement tool. However, this was not thought to be considerable enough to warrant a downgrade by any of the authors. Though the results contained some inconsistencies, we agreed they could be potentially explained by the use of different tasks, and all studies at least found some effect, so we did not downgrade. Four out of the six included studies in this outcome were direct (this outcome was the aim of their study), so evidence was not downgraded for directness either. However, a combination of poorly reported statistics, and small sample sizes (which all of them had), led us to downgrade for imprecision.

Depth

There was considered to be a small risk of bias in the measurements which tended also to use breathing belts, but with reasonable scores for control of confounding variables, and we all agreed there should be no downgrade. The results of pupil dynamics, however, were considerably inconsistent when considering them as a whole. The two studies that looked at changes in pupil fluctuations found an increase, but upon discussion we agreed to reduce the GRADE score because of the inconsistencies in the other aspects. We also downgraded for directness due to only two out of six articles having pupil and breathing as their main focus (those that looked at pupil fluctuations). Furthermore, the reported results were imprecise and/or insufficiently reported, leading to a unanimous downgrade, though it is worth noting that the two looking at pupil fluctuations were more precise.

Rate - direct

Here we split the direct and indirect studies because there were considerably more studies that addressed breathing rate. Of the direct studies, there was concern about the ratings for measurement robustness and control of confounding variables. One author thought this warranted a downgrade while the others did not. However, because there were serious and unanimous concerns about inconsistencies in the results, we downgraded for inconsistency, and did not downgrade on risk of bias, to err on the conservative side. While some included studies were indirect, there were thought to be enough direct ones (6/8), and we did not downgrade. However, there were a number of studies, including the more direct ones, that had poor reporting of statistics and small sample sizes which warranted a downgrade on imprecision.

Rate - indirect

Risk of bias here was also considered to be an edge case, with medium to low scores on ratings for measurement robustness and control of confounding variables. However, the following three categories again had more serious concerns, so risk of bias was left unchanged to be conservative. We downgraded for inconsistency as we could not adequately explain the variety of results. We downgraded for indirectness, as the relevant studies were almost exclusively indirect, and also for imprecision, as the results were again poorly reported for the comparison of interest (breathing rate and pupil dynamics).

In line with the GRADE guidance [67], we did not assess any outcome on the criteria for upgrading, because all outcomes had a low GRADE score after downgrading.
